# Supplementary material for: A Likelihood Approach for Real-Time Calibration of Stochastic Compartmental Epidemic Models
Source: PLoS Comput Biol. 2017 Jan 17;13(1):e1005257. doi: 10.1371/journal.pcbi.1005257 (PMC5240920; doi:10.1371/journal.pcbi.1005257)
Supplement: S1 File — (TAR.GZ) [file pcbi.1005257.s014.tar.gz › HSPH_Online-SI-Revision/IPoi09/n1-mild/n1-mild_BENCH2_table-8.pdf]

1	newly 3 weeks spec	14.	27.8519	{4., 64.}	135.714	0.422359	newly 3 weeks cumul	37.	66.2963	{10., 160.}	119.62	0.385172	newly 5 weeks spec	24.	35.3333	{9., 67.}	83.4877	0.344019	newly 5 weeks cumul	75.	133.981	{25., 283.}	113.951	0.368671	newly 63 weeks cumul	382.	668.13	{471., 814.}	83.5612	0.295734
2	newly 3 weeks spec	27.	45.4231	{13., 82.}	92.0228	0.288068	newly 3 weeks cumul	86.	157.192	{62., 244.}	93.9624	0.274947	newly 5 weeks spec	28.	28.3269	{7., 57.}	55.9753	0.293019	newly 5 weeks cumul	143.	219.442	{76., 354.}	72.9021	0.240509	newly 59 weeks cumul	317.	310.731	{105., 540.}	45.1225	0.22951
3	newly 3 weeks spec	15.	59.48	{0., 200.}	387.733	1.39297	newly 3 weeks cumul	45.	338.21	{0., 749.}	741.578	1.80394	newly 5 weeks spec	22.	7.26	{0., 17.}	89.6364	1.49469	newly 5 weeks cumul	87.	367.48	{0., 809.}	412.391	1.79543	newly 68 weeks cumul	303.	371.3	{0., 818.}	115.67	1.76501
4	newly 3 weeks spec	11.	36.5526	{11., 61.}	246.651	0.586083	newly 3 weeks cumul	28.	100.5	{22., 177.}	273.026	0.629329	newly 5 weeks spec	15.	37.8421	{11., 65.}	169.474	0.539992	newly 5 weeks cumul	54.	178.947	{39., 307.}	245.517	0.625477	newly 62 weeks cumul	437.	503.605	{165., 759.}	45.7003	0.371727
5	newly 3 weeks spec	17.	57.8387	{5., 100.}	267.173	0.713395	newly 3 weeks cumul	65.	182.645	{21., 286.}	204.615	0.666201	newly 5 weeks spec	31.	47.3871	{0., 108.}	117.17	0.772435	newly 5 weeks cumul	108.	286.387	{22., 482.}	190.681	0.679564	newly 64 weeks cumul	446.	432.613	{22., 839.}	54.4988	0.54455
6	newly 3 weeks spec	1.	14.87	{0., 47.}	1483.	0.896019	newly 3 weeks cumul	6.	24.67	{0., 71.}	416.833	0.983095	newly 5 weeks spec	7.	23.5	{0., 116.}	358.857	1.30394	newly 5 weeks cumul	16.	69.56	{0., 258.}	445.25	1.19069	newly 68 weeks cumul	409.	333.55	{0., 904.}	98.7946	1.82636
7	newly 3 weeks spec	31.	104.283	{0., 186.}	279.409	0.970036	newly 3 weeks cumul	65.	261.883	{0., 493.}	344.026	1.0482	newly 5 weeks spec	13.	74.8333	{0., 162.}	521.282	1.05532	newly 5 weeks cumul	100.	433.817	{0., 708.}	374.783	1.12773	newly 67 weeks cumul	468.	553.383	{0., 884.}	66.2642	0.860523
8	newly 3 weeks spec	7.	4.86957	{0., 14.}	85.0932	0.821005	newly 3 weeks cumul	20.	13.5652	{1., 52.}	80.8696	0.713532	newly 5 weeks spec	14.	4.95652	{0., 15.}	73.2919	1.08313	newly 5 weeks cumul	41.	23.5652	{1., 79.}	76.4581	0.791803	newly 59 weeks cumul	476.	218.043	{1., 534.}	59.0336	1.03434
9	newly 3 weeks spec	17.	39.5128	{12., 61.}	145.701	0.376385	newly 3 weeks cumul	37.	117.923	{60., 174.}	223.978	0.489984	newly 5 weeks spec	20.	27.8462	{7., 55.}	85.641	0.340176	newly 5 weeks cumul	77.	179.974	{77., 288.}	139.993	0.352688	newly 58 weeks cumul	379.	306.154	{132., 546.}	38.4209	0.221883
10	newly 3 weeks spec	34.	46.037	{17., 76.}	64.8148	0.286505	newly 3 weeks cumul	83.	147.352	{65., 225.}	91.0531	0.288025	newly 5 weeks spec	34.	35.037	{8., 63.}	50.5447	0.334883	newly 5 weeks cumul	149.	222.093	{102., 338.}	68.6677	0.251604	newly 59 weeks cumul	381.	415.167	{122., 699.}	44.9354	0.225277
11	newly 3 weeks spec	5.	10.8636	{0., 20.}	182.727	0.78718	newly 3 weeks cumul	14.	27.9545	{2., 69.}	155.519	0.551542	newly 5 weeks spec	14.	12.3636	{0., 27.}	66.8831	0.659157	newly 5 weeks cumul	35.	52.2727	{4., 112.}	113.506	0.512591	newly 58 weeks cumul	476.	296.727	{4., 637.}	51.0886	0.67862
12	newly 3 weeks spec	4.	14.7083	{2., 39.}	292.708	0.598856	newly 3 weeks cumul	11.	50.25	{10., 86.}	367.424	0.65157	newly 5 weeks spec	8.	15.2083	{0., 28.}	135.938	0.625907	newly 5 weeks cumul	23.	79.0417	{24., 145.}	256.703	0.570621	newly 59 weeks cumul	336.	199.292	{24., 447.}	55.7912	0.58003
13	newly 3 weeks spec	29.	25.9286	{5., 51.}	60.3448	0.418529	newly 3 weeks cumul	92.	102.667	{38., 178.}	53.2609	0.339845	newly 5 weeks spec	28.	12.8333	{0., 39.}	80.3571	0.860074	newly 5 weeks cumul	149.	135.833	{46., 270.}	49.8722	0.372109	newly 60 weeks cumul	314.	169.167	{49., 373.}	57.6054	0.536285
14	newly 3 weeks spec	28.	56.4237	{6., 98.}	131.174	0.416817	newly 3 weeks cumul	69.	119.373	{21., 226.}	108.278	0.385821	newly 5 weeks spec	32.	79.4407	{23., 120.}	164.248	0.445287	newly 5 weeks cumul	123.	269.051	{54., 447.}	142.111	0.416632	newly 64 weeks cumul	351.	782.305	{636., 908.}	126.993	0.364989
15	newly 3 weeks spec	16.	52.3871	{0., 106.}	261.29	0.758184	newly 3 weeks cumul	45.	152.968	{8., 272.}	272.616	0.75226	newly 5 weeks spec	24.	51.5484	{0., 121.}	179.301	0.862947	newly 5 weeks cumul	89.	260.032	{17., 490.}	224.792	0.722007	newly 64 weeks cumul	454.	468.935	{17., 870.}	63.0738	0.583343
16	newly 3 weeks spec	21.	44.7925	{11., 82.}	139.533	0.435892	newly 3 weeks cumul	39.	111.528	{26., 203.}	207.16	0.508772	newly 5 weeks spec	16.	56.434	{19., 91.}	265.448	0.562251	newly 5 weeks cumul	78.	217.377	{47., 373.}	196.009	0.496399	newly 62 weeks cumul	411.	721.509	{583., 838.}	80.3057	0.314783
17	newly 3 weeks spec	8.	38.3607	{0., 93.}	428.689	0.900025	newly 3 weeks cumul	19.	93.2131	{0., 230.}	435.116	0.949553	newly 5 weeks spec	11.	47.0984	{0., 89.}	368.703	0.920543	newly 5 weeks cumul	37.	183.672	{0., 409.}	436.907	1.0275	newly 65 weeks cumul	358.	605.525	{0., 840.}	105.207	0.893424
18	newly 3 weeks spec	10.	20.1071	{1., 43.}	135.357	0.473677	newly 3 weeks cumul	30.	58.5	{7., 111.}	119.048	0.353308	newly 5 weeks spec	13.	19.4286	{0., 42.}	103.297	0.567941	newly 5 weeks cumul	62.	96.5714	{15., 192.}	90.7834	0.347064	newly 58 weeks cumul	369.	241.464	{24., 459.}	49.758	0.45472
19	newly 3 weeks spec	26.	12.386	{0., 42.}	75.4386	1.03427	newly 3 weeks cumul	62.	27.1579	{0., 84.}	74.4199	0.97496	newly 5 weeks spec	40.	20.193	{0., 61.}	69.9561	1.06284	newly 5 weeks cumul	140.	62.9123	{0., 178.}	70.1253	1.04047	newly 65 weeks cumul	489.	519.86	{0., 862.}	62.5803	0.958359
20	newly 3 weeks spec	15.	18.36	{2., 44.}	76.2667	0.402775	newly 3 weeks cumul	42.	56.16	{10., 115.}	78.0952	0.315104	newly 5 weeks spec	12.	21.24	{0., 51.}	132.333	0.594659	newly 5 weeks cumul	79.	97.12	{11., 208.}	72.962	0.3327	newly 62 weeks cumul	337.	375.04	{48., 711.}	72.178	0.427085
21	newly 3 weeks spec	3.	2.58621	{0., 8.}	117.241	1.08542	newly 3 weeks cumul	24.	29.5517	{0., 95.}	122.27	1.07252	newly 5 weeks spec	14.	1.37931	{0., 3.}	98.5222	1.97178	newly 5 weeks cumul	48.	32.2759	{0., 123.}	88.9368	1.21407	newly 62 weeks cumul	267.	34.7241	{0., 123.}	86.9947	1.8116
22	newly 3 weeks spec	11.	19.0444	{2., 49.}	113.535	0.459788	newly 3 weeks cumul	21.	46.8889	{4., 109.}	155.661	0.529474	newly 5 weeks spec	8.	27.1556	{0., 60.}	272.222	0.68176	newly 5 weeks cumul	39.	96.4444	{21., 208.}	176.353	0.57091	newly 64 weeks cumul	470.	544.511	{23., 785.}	53.0449	0.551352
23	newly 3 weeks spec	17.	19.7708	{0., 49.}	81.9853	0.574639	newly 3 weeks cumul	40.	74.2292	{6., 161.}	126.406	0.507075	newly 5 weeks spec	12.	12.9167	{0., 32.}	80.5556	0.582625	newly 5 weeks cumul	68.	104.479	{6., 248.}	101.44	0.507388	newly 59 weeks cumul	373.	149.75	{6., 381.}	64.6113	0.786371
24	newly 3 weeks spec	11.	26.5	{0., 53.}	180.	0.645672	newly 3 weeks cumul	30.	69.675	{0., 153.}	172.417	0.657099	newly 5 weeks spec	19.	28.7	{0., 53.}	103.158	0.671021	newly 5 weeks cumul	65.	125.6	{0., 257.}	138.846	0.67951	newly 61 weeks cumul	445.	458.675	{0., 757.}	52.6685	0.651189
25	newly 3 weeks spec	8.	40.1951	{17., 65.}	408.537	0.698613	newly 3 weeks cumul	23.	116.659	{53., 177.}	411.877	0.688038	newly 5 weeks spec	6.	40.4146	{21., 61.}	578.455	0.837425	newly 5 weeks cumul	43.	197.634	{116., 276.}	364.379	0.669391	newly 58 weeks cumul	330.	493.829	{290., 679.}	60.0665	0.252287
26	newly 3 weeks spec	19.	42.6923	{13., 77.}	146.356	0.440631	newly 3 weeks cumul	55.	106.712	{21., 196.}	120.804	0.411323	newly 5 weeks spec	16.	52.5192	{15., 83.}	239.784	0.567069	newly 5 weeks cumul	94.	205.596	{54., 349.}	137.459	0.438964	newly 61 weeks cumul	295.	674.231	{487., 818.}	132.399	0.417143
27	newly 3 weeks spec	21.	16.4286	{2., 38.}	48.9796	0.376779	newly 3 weeks cumul	42.	44.0857	{5., 94.}	59.5238	0.327966	newly 5 weeks spec	24.	19.	{3., 35.}	48.2143	0.443955	newly 5 weeks cumul	84.	79.8857	{13., 169.}	51.9728	0.324842	newly 58 weeks cumul	327.	487.2	{282., 649.}	62.1844	0.271524
28	newly 3 weeks spec	3.	6.7381	{0., 18.}	215.079	0.867637	newly 3 weeks cumul	9.	16.6667	{0., 42.}	155.556	0.736414	newly 5 weeks spec	4.	7.21429	{0., 20.}	164.881	0.826132	newly 5 weeks cumul	17.	31.1429	{0., 78.}	156.583	0.822672	newly 64 weeks cumul	356.	284.143	{0., 668.}	72.4318	1.21729
29	newly 3 weeks spec	18.	29.8387	{1., 47.}	96.595	0.501506	newly 3 weeks cumul	43.	92.5806	{26., 154.}	134.359	0.42852	newly 5 weeks spec	35.	28.2903	{0., 54.}	45.8986	0.515924	newly 5 weeks cumul	101.	151.258	{31., 248.}	79.7189	0.380576	newly 61 weeks cumul	354.	349.419	{31., 674.}	54.9845	0.422373
30	newly 3 weeks spec	14.	38.5849	{3., 70.}	213.342	0.607416	newly 3 weeks cumul	49.	84.7925	{9., 146.}	120.485	0.528021	newly 5 weeks spec	21.	62.1132	{8., 108.}	219.677	0.621473	newly 5 weeks cumul	98.	199.283	{25., 352.}	140.085	0.56114	newly 65 weeks cumul	412.	715.943	{367., 891.}	91.8437	0.515117
31	newly 3 weeks spec	26.	54.3167	{10., 95.}	130.064	0.38138	newly 3 weeks cumul	72.	123.117	{19., 211.}	98.6806	0.347131	newly 5 weeks spec	44.	70.85	{24., 102.}	78.6742	0.283504	newly 5 weeks cumul	142.	257.483	{68., 402.}	99.331	0.324711	newly 63 weeks cumul	451.	804.883	{681., 891.}	80.0776	0.254546
32	newly 3 weeks spec	6.	19.8163	{0., 47.}	273.129	0.726331	newly 3 weeks cumul	17.	55.1429	{0., 127.}	264.226	0.722204	newly 5 weeks spec	8.	22.5714	{0., 51.}	229.592	0.713716	newly 5 weeks cumul	37.	98.8163	{0., 208.}	210.204	0.726328	newly 61 weeks cumul	420.	466.612	{0., 733.}	49.5724	0.637384
33	newly 3 weeks spec	16.	33.0222	{5., 62.}	138.889	0.504042	newly 3 weeks cumul	37.	84.5556	{8., 162.}	154.955	0.471887	newly 5 weeks spec	21.	41.7556	{8., 79.}	127.196	0.445404	newly 5 weeks cumul	77.	162.111	{32., 280.}	135.988	0.457559	newly 62 weeks cumul	388.	613.533	{301., 811.}	72.2738	0.349688
34	newly 3 weeks spec	7.	14.6	{0., 41.}	176.	0.650157	newly 3 weeks cumul	27.	57.4	{16., 117.}	142.222	0.383306	newly 5 weeks spec	14.	6.	{0., 19.}	76.	1.21108	newly 5 weeks cumul	53.	73.52	{16., 186.}	97.283	0.399595	newly 61 weeks cumul	269.	82.52	{16., 199.}	73.0409	0.776457
35	newly 3 weeks spec	10.	15.5306	{0., 37.}	109.592	0.52349	newly 3 weeks cumul	37.	36.9184	{2., 79.}	73.2488	0.495493	newly 5 weeks spec	22.	22.3469	{0., 51.}	73.5622	0.561732	newly 5 weeks cumul	79.	78.8163	{2., 181.}	70.9636	0.515871	newly 63 weeks cumul	462.	603.163	{2., 813.}	59.2323	0.517342
36	newly 3 weeks spec	18.	22.8462	{1., 53.}	87.7778	0.518148	newly 3 weeks cumul	39.	46.5231	{4., 106.}	82.4852	0.480365	newly 5 weeks spec	49.	42.0615	{4., 90.}	58.2418	0.531294	newly 5 weeks cumul	114.	120.523	{13., 270.}	68.8259	0.492875	newly 65 weeks cumul	404.	730.646	{123., 911.}	101.409	0.552302
37	newly 3 weeks spec	12.	8.23077	{0., 29.}	106.41	1.36846	newly 3 weeks cumul	26.	34.8077	{0., 72.}	114.941	0.745392	newly 5 weeks spec	13.	4.11538	{0., 13.}	98.5207	1.67064	newly 5 weeks cumul	55.	45.6538	{0., 122.}	90.6993	0.886508	newly 61 weeks cumul	310.	61.4231	{0., 151.}	88.6973	1.48315
38	newly 3 weeks spec	35.	48.7143	{1., 109.}	92.7551	0.578993	newly 3 weeks cumul	65.	106.75	{2., 252.}	115.165	0.612873	newly 5 weeks spec	24.	66.8214	{1., 112.}	208.036	0.666833	newly 5 weeks cumul	117.	232.661	{7., 462.}	137.408	0.641717	newly 65 weeks cumul	464.	682.286	{10., 876.}	73.9763	0.586444
39	newly 3 weeks spec	28.	15.28	{0., 40.}	80.8571	1.05788	newly 3 weeks cumul	72.	74.08	{0., 170.}	67.7778	0.620491	newly 5 weeks spec	31.	8.2	{0., 32.}	83.6129	1.69339	newly 5 weeks cumul	130.	92.12	{0., 226.}	65.8769	0.74273	newly 63 weeks cumul	377.	101.24	{0., 246.}	75.7984	1.09394
40	newly 3 weeks spec	16.	108.914	{34., 167.}	584.159	0.807491	newly 3 weeks cumul	56.	261.81	{52., 480.}	374.107	0.644046	newly 5 weeks spec	15.	98.1897	{38., 139.}	558.046	0.812696	newly 5 weeks cumul	83.	468.534	{212., 658.}	467.948	0.764282	newly 66 weeks cumul	397.	781.224	{569., 912.}	101.516	0.357592
41	newly 3 weeks spec	13.	21.7727	{0., 59.}	146.737	0.801809	newly 3 weeks cumul	28.	40.5455	{1., 98.}	115.584	0.647606	newly 5 weeks spec	36.	50.2727	{0., 137.}	112.205	0.844415	newly 5 weeks cumul	89.	126.606	{1., 312.}	113.858	0.729528	newly 67 weeks cumul	337.	658.742	{1., 927.}	135.136	0.811669
42	newly 3 weeks spec	5.	50.7593	{6., 99.}	933.704	1.00128	newly 3 weeks cumul	20.	97.2037	{11., 202.}	407.87	0.745521	newly 5 weeks spec	30.	88.5	{6., 153.}	221.79	0.682017	newly 5 weeks cumul	64.	258.519	{43., 454.}	325.058	0.756509	newly 66 weeks cumul	325.	752.889	{413., 923.}	150.154	0.65154
43	newly 3 weeks spec	39.	39.55	{0., 160.}	135.564	1.62112	newly 3 weeks cumul	61.	69.58	{0., 221.}	145.672	1.50242	newly 5 weeks spec	44.	45.9	{0., 170.}	127.318	1.58147	newly 5 weeks cumul	146.	164.49	{0., 649.}	139.829	1.64631	newly 68 weeks cumul	494.	372.64	{0., 908.}	81.7328	1.74578
44	newly 3 weeks spec	19.	27.5909	{0., 57.}	105.981	0.778204	newly 3 weeks cumul	73.	85.4545	{3., 171.}	79.5766	0.57502	newly 5 weeks spec	14.	20.4545	{0., 47.}	127.922	0.925578	newly 5 weeks cumul	103.	129.409	{3., 282.}	88.2171	0.630276	newly 60 weeks cumul	300.	223.182	{3., 520.}	66.8485	0.771623
45	newly 3 weeks spec	26.	26.9512	{6., 51.}	57.1295	0.374787	newly 3 weeks cumul	61.	92.8537	{21., 167.}	86.9252	0.36917	newly 5 weeks spec	23.	18.3415	{2., 34.}	53.9767	0.514951	newly 5 weeks cumul	105.	131.805	{32., 234.}	63.5308	0.333732	newly 58 weeks cumul	383.	218.854	{52., 385.}	47.6724	0.454017
46	newly 3 weeks spec	13.	8.70588	{0., 26.}	78.8839	0.97872	newly 3 weeks cumul	26.	21.451	{0., 61.}	81.5988	1.02281	newly 5 weeks spec	17.	12.3333	{0., 32.}	75.2018	1.02117	newly 5 weeks cumul	55.	44.2941	{0., 117.}	79.7861	1.0747	newly 65 weeks cumul	489.	324.745	{0., 728.}	63.527	1.39047
47	newly 3 weeks spec	7.	9.2973	{0., 26.}	111.583	0.704105	newly 3 weeks cumul	19.	23.2703	{0., 60.}	95.3058	0.612578	newly 5 weeks spec	6.	11.0541	{0., 34.}	170.721	0.956945	newly 5 weeks cumul	28.	45.027	{1., 114.}	127.027	0.655694	newly 63 weeks cumul	357.	381.378	{1., 733.}	75.0397	0.831128
48	newly 3 weeks spec	17.	42.	{21., 66.}	151.51	0.370647	newly 3 weeks cumul	40.	127.703	{58., 189.}	221.824	0.475713	newly 5 weeks spec	17.	34.5946	{18., 55.}	106.995	0.287425	newly 5 weeks cumul	72.	199.162	{91., 288.}	178.341	0.4166	newly 58 weeks cumul	326.	415.378	{200., 618.}	47.8776	0.18193
49	newly 3 weeks spec	2.	19.8421	{0., 41.}	928.947	1.02654	newly 3 weeks cumul	12.	53.4737	{1., 110.}	378.07	0.746281	newly 5 weeks spec	8.	26.	{0., 50.}	263.158	0.794533	newly 5 weeks cumul	27.	103.237	{1., 205.}	318.421	0.775646	newly 62 weeks cumul	311.	477.868	{1., 755.}	94.0684	0.706141
50	newly 3 weeks spec	19.	46.7458	{7., 75.}	164.05	0.461935	newly 3 weeks cumul	64.	119.814	{21., 196.}	107.071	0.347344	newly 5 weeks spec	20.	53.678	{23., 76.}	178.39	0.446392	newly 5 weeks cumul	105.	225.39	{88., 329.}	128.668	0.374115	newly 59 weeks cumul	406.	676.797	{564., 780.}	71.032	0.248626
